# Production and First Assessment of Iranian Secondary Tritipyrum Genotypes by GISH and AFLP Markers

**DOI:** 10.30498/IJB.2019.91760

**Published:** 2019-12-01

**Authors:** Hossein Shahsavand Hassani, Frank R. Blattner, Andreas Houben, Andreas Bӧrner

**Affiliations:** 1 Department of Agronomy and Plant breeding, College of Agriculture, Shahid Bahonar University of Kerman (SBUK), Kerman, Iran.; 2 Department of Crop Production and Plant Breeding, College of Agriculture, Shiraz University, Shiraz, Iran; 3 Genebank Department, Leibniz Institute of Plant Genetics and Crop Plant Research (IPK), Correns str. 3, D-06466, Gatersleben, Germany

**Keywords:** AFLP- GISH- ISTG- Non-Iranian Primary Tritipyrum- Thinopyrum bessarabicum- Wheat

## Abstract

**Background:**

Non-Iranian Primary Tritipyrum (2n=6x=42, AABBE^b^E^b^) set seed after Triticale (2n=6x=42, AABBRR) and Tritordeum (2n=6x=42, AABBH^c^H^c^) but, due to a few undesirable agronomic traits, it cannot fulfil the commercial expectations of farming.

**Objectives:**

To remove these deficiencies, six hexaploid Tritipyrum lines were crossed with four Iranian bread wheat cultivars which led to the production of 107 (F_1_), 479 (F_2_), 768 (F_3_), and 1539 (F_4_) Iranian Secondary Tritipyrum Genotypes (ISTG) seeds. This study was carried out for selecting the plants potentially carry the 5E^b^ chromosome/s and are good candidates for salt tolerant by GISH and RFLP markers.

**Materials and Methods:**

The procedure involved extracting the total DNA content of 209 plants, including non-Iranian primary Tritipyrum lines, Iranian wheat cultivars, Chinese Spring addition, and substitution lines for 5E^b^ and Iranian secondary Tritipyrum genotypes (ISTG: F_1_, F_2_, F_3_, F_4_). Genomic in situ Hybridization (GISH) on mitotic spreads of fertile new Iranian secondary Tritipyrum genotypes (ISTG) was carried out to demonstrate the feasibility of single E^b^ chromosomes. There were three trials of 18 Fragment Length Polymorphism (AFLP) EcoRI/MseI primers to identify the presence of the 5E^b^ chromosome in 105 ISTG plants, along with four wheat addition lines and substitution lines for the 5E^b^ chromosome.

**Results:**

GISH on mitotic spreads demonstrated the feasibility of producing 75 plants out of 105 fertile new Iranian secondary *Tritipyrum* genotypes (ISTG) with 0-14 single E^b^ chromosomes. Among the mentioned markers, only the E_36_/M_59_ marker showed 43, 50, 30 and 47 identical bands, respectively, in contrast to 53 expected bands in all plants with the 5E^b^ chromosome which indicated 21, 33, 9 and 6 out of 75 ISTG plants, respectively, with the 5E^b^ chromosome.

**Conclusion:**

This study indicated that 69 ISTG Tritipyrum plants were potentially carry the 5E^b^ chromosome/s and are good candidates for salt tolerant tests in comparison with Iranian modern bread wheat cultivars.

## 1. Background

Wild relatives of wheat offer a wide range of useful traits such as resistance to biotic and abiotic stresses ( 5 ). Despite being exploited extensively, the Triticeae wild relatives continue to be important sources of genes for introducing desirable agronomic traits into common and durum wheat ( 29 ). Thus, alien gene transfer into common wheat via cross-species hybridization increases the common wheat’s resistance to biotic and abiotic stresses and improves its quality ( 32 ). In this context, species in *Aegilops*, *Secale*, and *Thinopyrum* genera have been proven as valuable sources of new genes ( 31 ). The introgression of genetic variation from genus *Thinopyrum* species ( 6 , 12 ) into wheat has been practiced for more than 70 years ( 44 ), resulting in the transfer of 30 economically important traits ( 11 ). Many *Thinopyrum* amphiploids have been produced since the 1930’s ( 13 , 30 ) and have acquired perennial habits, along with tolerance to salt and drought ( 3 , 18 ).

Development of the durum wheat hybrids (*Triticum turgidum* L.) × *Thinopyrum bessarabicum* as new salt-tolerant *Tritipyrum* crops (AABBE^b^Eb) was first attempted in England using the diploid grass *T. bessarabicum*. The hybrids (*Tritipyrum* lines) had brittle rachis, poor agro type and thresh ability, which impeded progress in their breeding ( 14 ).

The introgression of *T. bessarabicum* chromosomes (2n=2x=14, E^b^Eb) *Triticum durum* (2n=4x=28, AABB) led to the transfer of new useful traits into primary *Tritipyrum* lines as well((2n=4x=28,AABBE^b^Eb) ( 34 - 35 ). Although E^b^ is recognized with high tolerance to 350 mM of NaCl, but primary *Tritipyrum* lines can set seed in 250 mM NaCl with few undesirable agronomic traits such as brittle rachis and late maturity ( 1 , 36 ). The 5E^b^ chromosome which carries most of the genes responsible for salt tolerance has been identified in wheat/alien recombinants ( 26 , 41 , 46 , 47 ). Although non-Iranian primary *Tritipyrum* lines have the potential to become a new salt tolerant cereal ( 15 , 19 , 33 , 37 , 39 , 42 ), they show brittle rachis and late maturity. Molecular cytogenetic techniques, such as fluorescent *in situ* hybridization (FISH) and genomic *in situ* hybridization (GISH), are excellent tools to analyze the genomic structure and function, chromosome constituents, recombination patterns, alien gene introgression, genome evolution, aneuploidy, polyploidy, as well as genome constitution, visualization and chromosome discrimination from different genomes in allopolyploids of various crops ( 10 , 2 , 45 ). FISH and its variants have been widely employed in karyotype characterization of plants ( 28 ). The technique is primarily based on the pairing of a given probe (DNA or RNA fragment/s) with a specific sequence on the target genome, aiming to indicate its exact location in a chromosome ( 17 ). GISH has been widely and successfully applied to identify parental genomes in hybrid cells ( 24 ), detecting alien segments in translocations and analyzing chromosome pairing ( 20 , 25 ), applying genetic improvement programs, evolution of polyploids, analyzing the meiotic behavior in hybrids, polyploids, and providing information concerning the relationship between species ( 43 ). Both these techniques become reliable for studying allopolyploids since most cultivated plants have been developed through hybridization or polyploidization. Hybrid derivatives may have variable alien chromosome numbers or chromosome arms, so the use of these approaches opens new avenues for an accurate identification of genomic differences ( 45 ). Chromosome dynamics have been observed in subsequent generations of hybrids (*Aegilops ovata × Secale cereal*: UUMMRR) during the mitotic metaphase of root meristems and the first metaphase of meiosis in pollen mother cells. According to the available scientific literature, chromosomes have been identified by GISH and FISH using pTa71, pTa791, and pSc119.2 and pAs1 DNA probes. The preferential transmission of chromosome 4M appeared during both androgenesis and gynogenesis. It is also hypothesized that the expression of the triticale Gc gene suppressor had an influence on the semi-fertility of hybrids but did not inhibit the chromosome rearrangements ( 21 , 23 ).

## 2. Objectives

The main aims of the present study were, firstly, to produce ISTG plants ( 38 , 39 , 40 ), i.e., *Tritipyrum* in which selected E^b^ chromosome/s is/are replaced by the D genome chromosomes of bread wheat and also characterize E^b^ chromosomes in the produced lines using GISH and AFLP techniques. Secondly, this research aimed at identifying specific ISTG plants (2n=6x=42, 7”A7”B6”D1”Eb) where 5E^b^ chromosome/s is/are replaced with 5D chromosomes.

## 3. Materials and Methods

### 3. 1. Production of ISTG Plants

Spikes of six hexaploid primary *Tritipyrum* lines (2n=6x=42, AABBE^b^Eb) were emasculated and were subsequently pollinated with four Iranian wheat cultivars. The F_1_ plants (2n=6x=42, AABB7′D7′Eb) were selfed for three consecutive growing seasons (Table 1).

**Table 1:** Seeds set and production of ISTG plants from crossing between primary *Tritipyrum* lines with Iranian bread wheat cultivars

Parents		F_1_ Seeds	ISTG seeds		
Primary tritipyrum lines (♀)	Wheat cultivars (♂)				
			F_2_	F_3_	F_4_
(Ka*/b)(Cr*/b)	Navid	12	54	130	219
La*/b	Navid	21	86	121	212
Ma*/b	Sefidkhosheh	17	63	108	228
Ma*/b	Falat	11	71	117	331
St*/b	Omid	20	98	106	256
(Ma/b)(Cr/b)	Navid	16	107	186	293
**Total plants**		107	479	768	1539

* Az=Aziziah, Ka=Karim, Cr=Creso, La=Langdon, Ma=Macoun, St=Stewart and b=*Thinopyrum* bessarabicum

### 3. 2. Mitotic Spreads

**Fig1.**
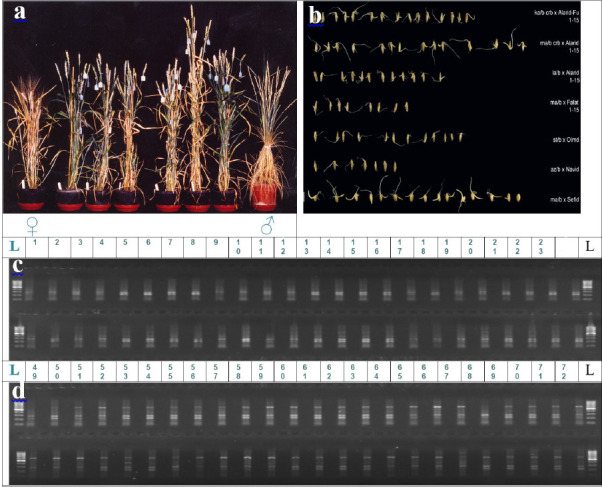
a) Non-Iranian primary “*Tritipyrum*” lines (middle; 6 plants), durum cultivar (far left plant) and *T. bessarabicum* species (far right plant). b) The germinated ISTG seeds from Non-Iranian primary *Tritipyrum*s (♀) with Iranian wheat cultivar (♂) crosses for GISH mitotic preparations from their roottips. c) The variation of AFLP polymorhpism of E36/M59 marker for non-Iranain primary “*Tritipyrum*” lines with E^b^ chromosomes in two above rows from 1 to 48 samples out of 209 samples (L=ladder). d) The variation of AFLP polymorphism of E36/M59 marker for Iranian secondary “*Tritipyrum*” genotypes (ISTG) with 5E^b^ chromosomes in two below rows from 49 to 64 samples (with 2 ladder) out of 209 samples.

When the ISTG seeds (Table 1) had germinated (Fig. 1b) and the roots measured one cm in length, they were excised and immersed in ice cold water for 24h. The roots were fixed in a solution having a ratio of 3 ethanol: 1 acetic acid before being squashed. The cover slips were removed after freezing in liquid nitrogen and the slides were air-dried for GISH as described by Mirzaghaderi ( 27 ) and Endo ( 8 ).

### 3. 3. Meiotic Spreads

Immature anthers were squashed in a drop of 45% acetic acid on a slide with gentle heating ( 34 ). The cover slips were removed after freezing in liquid nitrogen. The slides were dried by gentle heating and then selected by phase contrast microscopy.

### 3. 4. DNA Extraction

Genomic DNA was isolated from *T. bessarabicum* species and was sheared to 500bp pieces by sonication. The DNA was subsequently labeled with dig-11- dUTP using nick translation (Roche applied Science Company). Total genomic DNA from the wheat cultivar Chinese Spring (CS) was autoclaved for 5 min to give fragments of 100-500bp. The fragments were then used at different concentrations as blocking DNA. The total DNA of five non-Iranian primary *Tritipyrum* lines, four Iranian wheat cultivars, four CS addition lines, and four substitution lines for 5E^b^, ISTG plants (F_1_, F_2_ , F_3_ , F_4_ ) were extracted as described by Chen ( 4 ).

### 3. 5. GISH Experiment

**Fig2.**
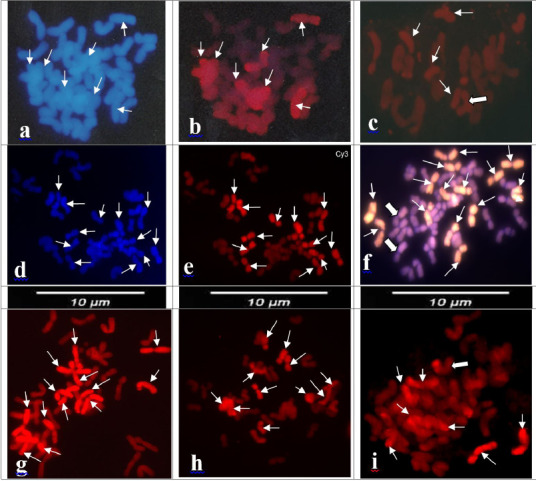
GISH on mitotic root tip cells of ISTG genotypes (F_1_, F_2_, F_3_, F_4_) and meiotic spreads of ISTG (F_2_) probed with E^b^ genome and blocked with bread wheat genomic DNA from CS. a) Mitotic cell of La/b×Navid F_1_ plant (2n=6x=42, AABBDE^b^) (blue). b) The same mitotic cell of F_1_ plants with 7 single Eb chromosomes (bright red, arrowed). c) GISH on meiotic cell of ISTG plants (Ma/b×Flat: F_3_) with 6 Eb chromosomes showing 4 single and 1 rod of Eb chromosomes (bright, red, arrowheads). d) Mitotic cell of ISTG plants (St/b×Omid: F_4_) (blue). e) GISH on the same mitotic cell of ISTG plants (St/b×Omid: F_4_) with 12 single E^b^ chromosomes (red, arrowed). f) The mitotic cell of ISTG (F_2_; (Ma/b)(Cr/b)×Navid) indicating 14 single E^b^ chromosomes (bright purple, arrowed) and two translocation chromosomes between Eb and A/B/D chromosomes (bright-dark: thick arrowheads). g) GISH on mitotic cell of ISTG plants (Ma/b)(Cr/b)×Omid: F_2_) with 13 single Eb chromosomes (red, arrowed) h) GISH on mitotic cell of ISTG plants (Ma/b×Sefidkhoshe: F_4_) with 11 single E^b^ chromosomes (red, arrowed) i) GISH on mitotic cell of ISTG plants (Ma/b×Flat: F_3_) with 7 single E^b^ chromosomes (red, arrowed) translocation chromosomes between E^b^ and A/B/D chromosomes (bright-dark: thick arrowheads).

The modified GISH technique was performed on ISTG plants as described by Shahsavand Hassani ( 21 ) and Endo ( 9 ). Mitotic and meiotic slides of ISTG plants were incubated in 100 µL of 2% PFA solution for 5 min, and were washed three times using 1xPBS for 5 min. The slides were dehydrated in ethanol series (70%, 90% and 100%) for 5 min each. Then, they were air-dried and denatured in 70% formamaide in 2xSSC mM phosphate buffer, pH 7, by being incubated on a heater (70 °C) for 2 min. This was followed by dehydration in ice-cold ethanol series for 3 min and air-drying. Labelled genomic DNA of *T. bessarabicum* and unlabeled CS blocking DNA were denatured by heating the hybridization solution at 90 °C for 7 min. The solution contained 70% (v/v) deionized formamaide and 50% dextrin sulfate (w/v) in 2xSSC. GISH was carried out on sealed slides with fixogum that were kept overnight at 37 °C in a dark and moist chamber oven. The hybridization mixture for each slide consisted of the E^b^ labeled genome as the probe (10µL), deionized formamaide (1.2-2)µL, sterile 20xSSC (5µL), 50% Dextrin sulphate (1µL), and blocking DNA (2.8 µL). After hybridization, the fixogum was taken off and the slides were washed three times in 2xSSC for 5 minutes by gentle shaking. Subsequently, 100 µL of 1% (w/v) blocking reagent belonging to the antibody mixture solution in 2xSSC (99 µL donkey Rodamin antibody + 1 µL sheep Rodamin antibody for each slide) was added with paraffin cover slip. The solution was then incubated in a dark-moist oven at 37 °C for 1 hour. On each slide, 14µL of antifade DAPI (1µg.-µL stock) was positioned with cover slip and visualized by Olympus microscope. The GISH of E^b^ genome chromosomes (Fig. 2_a-i_) were obtained from the negative pictures after conversion to gray scale.

### 3. 6. AFLP Experiment

The genomic DNA of 209 plants (Table 2) were extracted as described by Chen ( 4 ) and the AFLP protocol was conducted as described by Blattner ( 38 ).

**Table 2:** ISTG progenies evaluated for the presence of 5Eb chromosome along with the control parental, additions and substitution lines

Wheat/5E^b^ addition lines		Wheat 5E^b^ substitution lines	E^b^ accessions	Wheat cultivars (♂)	Primary tritipyrum (♀)	ISTG progenies
CS/5E^b^		CS(5A)5E^b^	1339	Navid	(Ma/b)(Cr/b)	(Ma/b)(Cr/b)×Navid(F_1_)
CS/5E^b^S		CS(5A)5E^b^L	10232	Omid	St/b	St/b×Omid(F_3_)
Genaro/5E^b^		CS(5D)5E^b^	53171	Flat	Ma/b	Ma/b×Flat(F_2_)
8x.tritipyrum (CS/E^b^)		CS(5D)5E^b^L	21890	Sefid Khoshe	Ma/b	Ma/b×Sefidkhoshe (F_3_)
---------------		------------	------------	Navid	La/b	La/b×Navid(F_4_)
----------------		------------	------------	Navid	(Ka/b)(Cr/b)	(Ka/b)(Cr/b)×Navid(F_4_)
**Total plants**	16	23	4	46	15	105

### 3. 6. 1.Restriction Ligation

An amount of 0.2 μg genomic DNA (Table 2) was digested with MseI and EcoRI enzymes. Five pmol EcoRI and 50 pmol MseI adaptors were ligated with 1 U T4 DNA ligase in a buffer containing 10 mM Tris- HAc pH 7.5, 10 mM MgAc, 50 mM KAc, 5 mM DTT,1mM ATP and 50 ng.L^-1^ bovine serum albumine in a

total volume of 11 μL for 3h at 37 °C.

### 3. 6. 2. Preselective Amplification

PCR was set in 4 μL diluted restriction ligation DNA, 2.5 pmol EcoRI and 2.5 pmol MseI, respectively, along with 0.4 U Taq DNA polymerase (Qiagen GmbH), in addition to 0.2 mM of each dNTP (Amersham- Pharmacia Biotech) and 1x Qiagen PCR buffer in a volume of 20 μL. The PCR reactions were performed in a PE 9600 thermal cycler programmed for 20 cycles at 94 °C (1s), 56 °C (30s), 72 °C (2 min). To verify success in amplification, 10 μL of the PCR mixture was electrophoresed on a 1.5% agarose gel in 1xTAE buffer stained with 0.5 μg.-mL ethidium bromide.

### 3. 6. 3.Selective Amplification 

For amplification, one cycle was performed for 30s at 94 °C, 30s at 65 °C and 2 min at 72 °C. This was followed by 8 cycles in which annealing temperature was subsequently lowered by 1° C per cycle, and 23 cycles of 1s at 94 °C, 30s at 56 °C and 2 min at 72 °C. For sample loading, 2 μL of the PCR product was mixed with 0.15 μL of 6-carboxy- X-rhodamin (ROX)-labeled internal length standard GeneScan-500 ROX and 0.85 μL formamaide dye. It was denatured for 3 min at 90 °C and then chilled on ice.

Electrophoresis of DNA was carried out using 5% denaturing polyacrylamide gels in 1xTBE electrophoresis buffer. AFLP fragments were analyzed using Mega base analysis software (Applied Biosystems).

Survey of phenotypic pools for AFLP polymorphism was carried out with 16 *Eco*RI/*MseI* primer combinations (Table 3). The polymorphic bands were scored as either the presence or absence of 5E^b^ chromosomes in ISTG plants (Table 5).

**Table 3:** The specific AFLP enzyme combinations of 5E^b^ chromosome in *T. bessarabicum*

AFLP enzyme combinations	First trail	Second trail	Third trial
E_35_/M_50_			
E_36_M_47_(214,320,325)bp	E_35_/M_50_	E_36_/M_59_	E_36_/M_59_
E_36_M_59_(149, 152,198,260,420)bp	E_36_/M_59_	E_36_/M_60_	
E_36_M_60_(227)bp	E_36_/M_60_	E_36_/M_62_	
E_36_M_61_(89,113,189,360,464)bp	E_36_/M_61_		
E_36_M_62_(191,210)bp	E_36_/M_62_		
E_37_M_47_(182,395)bp	E_40_/M_61_		
E_37_M_59_(155,323,398)bp	E_41_/M_49_		
E_37_M_60_(182,277,283)bp	E_41_/M_61_		
E_37_M_61_(117,410)bp			
E_37_M_62_(167,169,180,416,423)bp			
E_41_/M_49_			
E_40_M_50_(138bp)			
E_40_/M_61_			
E_40_/M_61_			
Total: 15	8	3	1

## 4. Results

ISTG seeds (2n=6x=42, AABB6″D1″E^b^) were obtained from selfing the F_1_ plants (2n=6x=42, AABB7′D7′Eb), 479(F_2_), 768(F_3_) and 1539(F_4_) (Table 1). The success indicates a high level of crossability between parents with 1E^b^ to 7E^b^ chromosomes, in addition to a satisfactory level of fertility in F_2_, F_3_, and F_4_ progenies (Table 1). These results are in agreement with Shahsavand Hassani et al., who found a high degree of crossability and fertility in segregation generations ( 34 , 36 , 37 ).

GISH on F_1_ mitotic preparations (Fig. 1a-b) showed 7 single E^b^ chromosomes. The meiotic (Fig. 2c) and mitotic preparations of ISTG plants (Table 4 and Fig. 2d-i) identified 0-14 E^b^ chromosomes. The GISH-E^b^ probe detected the alien segments of E^b^ chromosomes in ISTG plants, e.g. the occurrence of Robertsonian translocation which enabled the exchange between the short arm of E^b^ chromosomes and the long arm of A, B, and D chromosomes (Fig. 2, thick arrowhead). This is also in agreement with previous results reported by Shahsavand Hassani et al. (34).

**Table 4:** Identification of E^b^ chromosomes in ISTG plants by GISH method using genomic E^b^ probe.

ISTG Genotypes	Plant Number	Cell Number	2n	Number of single E^b^ chromosomes
La/b × Navid (F_4_)	13	20	42	4-5
(Ka/b)(Cr/b) × Navid (F_4_)	16	52	42	6-14
(Ma/b)(Cr/b) × Navid (F_1_)	2	4	42	7
Ma/b × Flat (F_2_)	11	26	42	0-10
Ma/b × sefidkhoshe (F_3_)	18	24	42	4-8
St/b × Omid (F_3_)	6	16	42	12
Total	68	142	42	0-14

Although the A, B, and D genomes have been identified by GISH using a ratio of 80:1 (blocking + probes), a good discrimination among wheat genomes was obtained with the ratio of 100:1 between the blocking and probes. This appropriate discrimination is due to a high similarity between E^b^ genomes and the three wheat genomes (Fig. 2). The E^b^ chromosomes in ISTG hybrids were distinguished by using digoxigenin labeled DNA from A, B, and D genomes (Fig. 2 and Table 4). The fluorescence hybridization signals were homogeneously distributed along the chromosomes and the E^b^ chromosomes were visualized as red. The chromosomes of A, B and D genomes were faint brown due to a slight amount of cross-hybridization (Fig. 2b,c,e-i)

Variations existed among ratio bands that were observed and expected of 5E^b^ chromosomes with AFLP primers in 75 plants of ISTG, either with or without the 5E^b^ chromosome (Table 5 and Fig. 1c-d). AFLP markers for 5E^b^ chromosomes in 47 of ISTG plants revealed segments of the E^b^ genome with the size of a single marker, which probably resulted from recombination between the wheat and the wild E^b^ chromosomes (Table 3 and Fig. 1d).

The results of GISH herein were based on the E^b^ genomic DNA probe. Together with the results pertaining to 16 RFLP DNA markers of the 5E^b^ chromosome (Table 3), these findings will further encourage research on wheat-*Thinopyrum*-wide hybridization by signaling ISTG germplasm. The approach can be regarded as a new potential substitution in relation to salt-tolerant *Tritipyrum* genotypes for the improvement of wheat.

Seventy five ISTG plants were effectively screened using GISH images (Fig. 2 and Table 4) which present a general overview of the genome in the hybrid plants. Meanwhile, the AFLP analysis reveals that the genetic identity of the alien chromosomes and chromosomal segments introgressed in 47 of ISTG plants containing the 5E^b^ chromosome. Eight AFLP EcoRI/MseI combinations (Table 3) had almost the same bands in the 5E^b^ chromosome. Eight AFLP EcoRI/MseI combinations (Table 3) had almost the same bands in CS additions and substitution lines regarding the 5E^b^ chromosome. Three specific AFLP markers showed numerous bands in ISTG plants (Table 3). The specific 5E^b^ fragments (i.e. 149-152bp of E_36_/M_59_) showed 43 and 50 identical bands, respectively (Table 5).

**Table 5:** The AFLP polymorphism of E_36_M_59_ fragments in substitution ISTG plants for 5E^b^ chromosome.

E_36_M_59_ Fragments (bp)	Plant Materials	Genomic DNA	Bands	
			Present	absent
	With 5E^b^			
149	AABBE^b^E^b^	36	5	31
	E^b^E^b^	10	0	10
	(CS/5E^b^)	16	14	2
	[CS/(5E^b^/5A,B,D)]	18	17	1
	AABBDDE^b^E^b^	8	4	4
	Without 5E^b^			
	AABBDD	8	5	3
	*AABB*	3	3	0
	ISTGs	35	21	14
	Observed bands	43	43	51
	Expected bands	53	53	46
	With 5E^b^			
152	AABBE^b^E^b^	36	30	6
	E^b^E^b^	10	8	2
	CS/5E^b^	16	16	0
	[CS/(5E^b^/5A,B,D)]	18	18	0
	AABBDDE^b^E^b^	8	8	0
	Without 5E^b^			
	AABBDD	8	6	2
	*AABB*	3	2	1
	ISTGs	35	33	2
	Observed bands	50	50	11
	Expected bands	53	53	46

## 5. Discussion

The GISH was able to identify alien E^b^ chromosome/s which introgressed into ISTG genotypes by mitotic and meiotic spreads (Fig. 2). This is in agreement with Shahsavand Hassani et al. ( 7 , 16 , 34 - 35 ). 

The GISH-E^b^ genomic probe on 75 of ISTG plants showed a range of 0-14 E^b^ chromosomes (Table 4). Even though these 75 plants have the potential to be named as Iranian secondary *Tritipyrum* genotypes, the GISH technique was not able to differentiate the 5E^b^ chromosome from the other E^b^ chromosomes (Table 2). The inability could be due to the cross hybridization of the E^b^ genomic probe with A, B, and D chromosomes (Table 4), as well as the existence of a genetic similarity with the E^b^ chromosomes of *T. bessarabicum* species. In a relevant study, four diploid and two tetraploid *Aegilops* species were analyzed, along with three *Aegilops* × *Secale* hybrids. The analyses involved the use of FISH with pSc119.2, pAs1, 5S rDNA, and 25S rDNA clones to differentiate the U, M, S, and D sub genome chromosomes of the *Aegilops* genus. Differences in the hybridization patterns by GISH allowed to identify all U, M, S, and D sub genome chromosomes. Some differences were detected in the localization of rDNA, pSc119.2 and pAs1 sequences between analogue sub genomes in diploid and tetraploid species and *Aegilops* × *Secale* hybrids. The hybridization pattern of the M and S genomes was more variable than that of the U and D genome ( 22 , 23 ). Therefore, it might be useful to further analyze these ISTG genotypes with pSc119.2, pAs1, 5S rDNA, and 25S rDNA clones as probes in a FISH analysis to differentiate between the A, B, E^b^ and D genome chromosomes. 

The specific AFLP marker for chromosome 5E^b^ facilitated the characterization of the Iranian bread wheat substitution lines when considering this chromosome in 47 of ISTG plants (Table 5). The circumstances were associated with enough seeds which potentially belong to the new Iranian secondary salt-tolerant *Tritipyrum* genotypes that bear A, B, D and E^b^ genomes. 

The GISH and AFLP succeeded in yielding results that illustrate the feasibility of chromosome differentiation in relation to the E^b^ genome within the non-Iranian primary and the Iranian secondary *Tritipyrum* genotypes (Tables 2, 3, 4, 5 and Fig. 1c-d). These findings can be read consistently and parallel to previous reports by Zhang et al. ( 46 ) and Shahsavand Hassani et al. ( 37 ).
